# Combined effect of acute salt and nitrogen stress on the physiology of lichen symbiotic partners

**DOI:** 10.1007/s11356-022-24115-0

**Published:** 2022-11-18

**Authors:** Karolina Chowaniec, Anna Żukowska-Trebunia, Kaja Rola

**Affiliations:** Institute of Botany, Faculty of Biology, Jagiellonian University, Gronostajowa 3, 30-387 Kraków, Poland

**Keywords:** Cell membrane integrity, Chlorophyll fluorescence, Lichens, Nitrogen excess, PSII maximal quantum yield, Salt stress

## Abstract

Nitrogen pollution and excessive salinity are commonly regarded as one of the major environmental concerns in recent decades in many urban environments. Although in urban areas lichens are exposed to both salt and nitrogen stress, no studies have been conducted to date on the simultaneous impact and interaction of these factors on lichen physiology. The aim was to determine the effect of various combinations of NaCl and NH_4_NO_3_ doses on the physiology of epigeic lichen *Cladonia rei*. We also aimed to compare the response of lichens collected from polluted and unpolluted sites to verify whether lichens exposed to different levels of environmental stress in their native environment will react differently. The combined salt-nitrogen treatment caused significant disturbances in the integrity of cell membranes and chlorophyll fluorescence parameters. The most detrimental effect concerned the loss of cell membrane integrity, which suggests that this parameter can serve as a relevant indicator of acute salt-nitrogen stress incidents. Salt stress decreased the photosynthetic efficiency 1 h after exposure, but after 72 h, the *F*_*V*_*/F*_*M*_ returned to the level characteristic of healthy lichens in experimental groups without and with small doses of ammonium nitrate. In contrast, recovery was not possible in combination with high nitrogen doses. This indicates that exposure to short-term salt stress in a nitrogen-poor environment only causes a temporary reduction in photosynthetic efficiency, but in urban eutrophic environments may have more serious consequences. The weakened physiological condition of the mycobiont manifested by an increased level of cell membrane damage and a persistent decrease in the photosynthetic efficiency of the photobiont in lichens growing along the roads may indicate an excess of nitrogen in the environment, enhanced by the effect of salt. Lichens collected from a heavy-metal-polluted habitat responded more strongly than those from an unpolluted habitat suggesting that in lichens previously affected by certain harmful factors, exposure to another stress factor may lead to greater disturbances. This is of particular importance for lichens inhabiting the vicinity of roads, since they are also under the influence of other pollutants emitted by road traffic.

**Supplementary Information:**

The online version contains supplementary material available at 10.1007/s11356-022-24115-0.

## Introduction

Urban pollution is a current problem in many cities worldwide, especially in highly urbanised areas. Pollution of highly populated areas may arise from various sources, but the most detrimental are those emissions related to human activities. Various pollutants are derived mainly from motor vehicles, industrial plants, combustion, and heating plants (Cohen et al. 2004). Nitrogen pollution is commonly considered one of the major environmental concerns related to anthropogenic activities in the last decades (Galloway et al. 1995; Erisman et al. 2007). The causes of increased pollution with nitrogen compounds may be the widespread use of vehicles, urban centres, photochemical smog or agricultural activities. These sources provide various forms of nitrogen, e.g., NO, NO_2_, and NH_3_ (Engardt et al. 2017). Due to the significant share of transport and industry, urban centers are important point sources of nitrogen pollution (Pataki et al. 2006). Traffic is a major source of nitrogen oxides, especially in areas with high traffic density. For example, the study conducted in the Boston area concerning the source of atmospheric N inputs showed that vehicles produced NO_X_ and NH_3_, which are the primary source of N deposition (Frati et al. 2006; Decina et al. 2017). Decina et al. (2019) reported that cities are hotspots of N deposition, and N enhancement is about twice higher in urban areas, regardless of their size and topography, than nearby rural sites. Another problem associated with urbanised areas is road salting during winter seasons. In many countries, NaCl is used for road de-icing, which disrupts infrastructure and the environment, especially in areas with a dense road network (Novotny et al. 2008). Many studies showed that urban areas are important sources of excessive salinity (Baker et al. 2004; Miyamoto and Chacon 2006; Laceby et al. 2019). Moreover, even after de-icing has been discontinued, high salt concentrations still occur in urban ecosystems for several years (Ludwikowski and Peterson 2018; Jamshidi et al. 2020). Data show that in Toronto, only 45% of Cl^−^ was removed yearly by surface runoff before the following winter (Howard and Haynes 1993). Cunningham et al. (2008) observed the highest accumulation of ions near roadways or downhill from parking lots even in autumn before winter salt application.

Both the problem of nitrogen excess and high salinity appear in urbanized areas. This certainly has a very negative impact on various components of the environment and disturbs the functioning of many organisms, including plants and lichens. Lichens constitute a symbiotic association between a heterotrophic fungus and autotrophic algae, and/or cyanobacteria. They are ectohydric organisms, which due to the lack of cuticles and roots, are particularly sensitive to the harmful impact of various toxic substances present in the environment (Ahmadjian 1993; Nimis and Purvis 2002). In the last century, SO_2_ was the main harmful air pollutant with a negative impact on the physiology of lichens. In recent years, however, nitrogen compounds have been recognized as a key factor affecting epiphytic lichens (Munzi et al. 2009; Van Dobben and de Bakker 1996). The most noticeable effect of nitrogen pollution concerns an increase in nitrophytic species and a decrease in acidophytic or neutro-nitrophytic ones (e.g., Frati et al. 2008; Paoli et al. 2013). In urban areas or in the vicinity of motorways, the positive correlation between traffic density and the level of nitrogen accumulation in lichen thalli has been confirmed in several studies (e.g., Gombert et al. 2003; Sujetovienė 2010). The uptake rate of various N forms depends on lichen species, the availability of N sources in the habitat, the type of substrate on which a lichen grows and the type of photobiont (Dahlman et al. 2004). Epiphytic lichens had significantly higher nitrogen and ammonium uptake than terricolous ones (Dahlman et al. 2004). Mycobiont is mainly responsible for the uptake of most of the N, which is deposited in the hyphal apoplastic continuum of the thallus, but the photosynthetic partner may nevertheless compete for N with the fungal partner (Dahlman et al. 2004). The effect of nitrogen excess on lichen vitality is not only species-specific but also time-and dose-dependent (Munzi et al. 2010). Various ecophysiological studies showed that both nitrate (NO_3_^−^) and ammonium (NH_4_^+^) forms of nitrogen could cause significant changes in the physiology of lichens, for example, induce cell membrane damage, membrane lipid peroxidation, and cause a decrease in photosynthetic efficiency as well as chlorophyll *a* and ergosterol contents (Gaio-Oliveira et al. 2004; Munzi et al. 2009, 2010; Paoli et al. 2010, 2015). The influence of other factors like solar radiation may increase the negative effect of nitrogen on lichens (Morillas et al. 2021). Nevertheless, nitrophytic and non-nitrophytic lichens differ in their response (e.g., Nybakken et al. 2009; Paoli et al. 2015; Wang et al. 2019a).

In the last two decades, a significant increase in sodium and chloride ions concentration has been observed in urban environments (Godwin et al. 2003; Cunningham et al. 2008), which certainly affects the vitality of lichens growing in cities. Elevated salt concentrations in the environment lead to a decrease in hydric potential affecting water availability in lichens (Hasegawa et al. 2000). The exposure to dehydration leads to a gradual loss of photosynthetic activities, which is manifested by the reduction of the maximum efficiency of PSII (e.g., Matos et al. 2011; Chowaniec and Rola 2022). Prolonged periods of metabolic inactivity in desiccated state can lead to a significant carbon loss and reduced lichen vitality (Mishler and Oliver 2018; Morillas et al. 2022). The hyperosmotic shock and accumulation of saline elements in the thallus may cause significant ion imbalances that induce the production of reactive oxygen species and contribute to cell membrane damage, reduction of protein or enzyme activity, and other metabolic disorders of both symbiotic partners (Erdmann and Hagemann 2001; Delmail et al. 2013; Yemets et al. 2015). Therefore, lichens are exposed to severe stress induced by increased salinity.

The vast majority of studies concern nitrogen or salt’s effect on epiphytic lichens, while little is known about the response of epigeic lichens. Both nitrogen excess and high salinity are common in urban environments, especially along communication routes. Nevertheless, to the best of our knowledge, there are no studies on the interaction of these two factors with lichen physiology. However, lichens are exposed to their simultaneous effects in urban environments. The study aimed to determine the effect of various combinations of sodium chloride and ammonium nitrate doses on the physiology of epigeic lichen *Cladonia rei* occurring in urban habitats. We also aimed to compare the effect of these compounds on the vitality of lichens collected from polluted and unpolluted sites to verify whether lichens exposed in their native environment to different levels of environmental stress will react differently. The following hypotheses were set: (1) combinations of ammonium nitrate and sodium chloride will show a more harmful effect on the lichen physiology than their individual effect; (2) lichens from polluted habitats will be more affected by combined salt and nitrogen stress than those from unpolluted habitats.

## Materials and methods

### Selected species

*Cladonia rei* Schaer. was selected for the study. It is a terrestrial lichen that occurs in dry and sunny habitats, such as heathlands, grasslands, forest edges or ruderal habitats (James 2009). The species also inhabits various anthropogenic habitats and is a dominant species in a cryptogamic community, *Cladonietum rei*, which is characteristic of disturbed sites in Europe (Paus 1997; Osyczka and Rola 2013; Rola et al. 2014; Rola and Osyczka 2018). This lichen frequently colonises soils contaminated with heavy metals and has been recognized as a pioneer species on post-industrial sites (Osyczka and Rola 2013).

The species has squamulose primary thallus and elongated podetia (secondary thallus) that are covered with granules and soredia in the upper part (Nimis 2016). It is characterized by high phenotypic plasticity with specific morphotype reported from highly polluted habitats (Osyczka et al. 2014).

*Cladonia rei* is typically associated with photosynthetic partners representing green algae of the *Asterochloris* genus; however, under unfavorable habitat conditions at heavy-metal-polluted sites, some individuals proved to harbor *Trebouxia* sp. representatives instead of a typical *Asterochloris* photobiont (Osyczka et al. 2021).

### Sample collection and handling

Lichen samples were collected in the autumn season in 2021. The samples originated from two study sites differing in heavy-metal pollution levels. The first study site constituted a post-smelting dump formed after the processing of zinc and lead ores in Trzebinia town in S Poland (hereinafter referred to as the polluted site). The second constituted a dry grassland community in Bukowno town (hereinafter referred to as the unpolluted site) (see Table 1 for details). The selection of sites was consistent with the assumption of using thalli from two extremely different habitats for the experiment to verify whether the response to the tested factors will differ between lichens exposed to varying levels of environmental stress in their native environment.

**Table 1:** The details of study sites with characteristics of chemical parameters of the soil substrate (mean ± SD; *n* = 10 for heavy-metal concentrations; *n* = 3 for C_org_, N_tot_ and pH). The data were retrieved from Osyczka and Rola (2019) and Rola and Osyczka (2018)

Location		Trzebinia town (polluted site)	Bukowno town (unpolluted site)
Coordinates		50° 09′ 13.6′′ N 19° 27′ 49.2′′ E	50° 15′ 21.7′′ N 19° 29′ 57.7′′ E
Habitat type		Post-smelting slag dump	Semi-natural grassland
Soil chemical parameters	Zn (mg/kg)	38 836.23 ± 19 224.34	80.72 ± 27.64
	Pb (mg/kg)	7126.65 ± 4654.78	13.24 ± 1.73
	Cd (mg/kg)	61.68 ± 41.95	0.26 ± 0.03
	As (mg/kg)	2846.80 ± 1584.97	0.91 ± 0.26
	Cu (mg/kg)	235.00 ± 77.82	11.88 ± 11.94
	Ni (mg/kg)	72.90 ± 13.81	30.44 ± 12.97
	C_org._ (%)	1.141 ± 0.14	0.801 ± 0.45
	N_tot._ (%)	0.070 ± 0.01	0.046 ± 0.03
	pH	6.55 ± 0.17	4.13 ± 0.07

The collected thalli were packed in paper envelopes with access to air. The thalli were cleaned of soil, plant, and bryophytes in the laboratory with tweezers and a brush.

### Experimental design

**Fig. 1:**
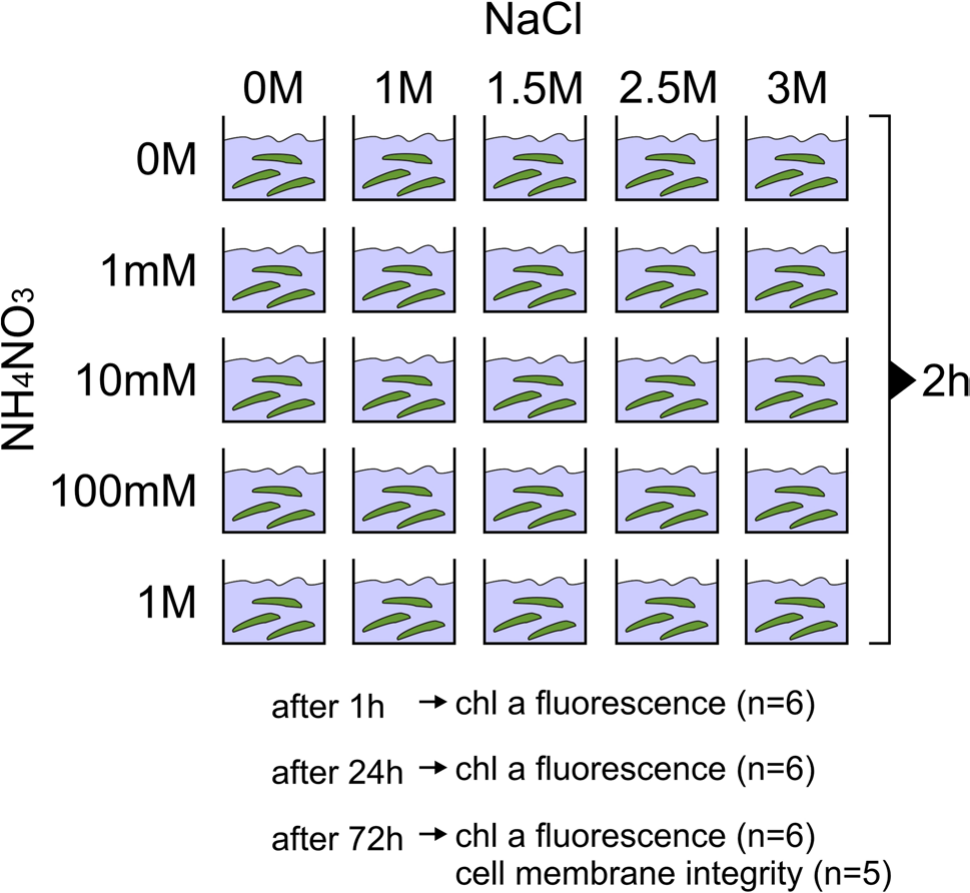
The design of the experiment. Number of replicates for particular analyses is provided

The experiments were carried out from November to December 2021. Only the secondary thallus (podetia) was intended for the experiment. Before the experiments, the lichen thalli were irrigated and kept for 48 h in a chamber with a relative humidity of 95% at laboratory room temperature (20 °C) at low light intensity (approx. 10 μmol photons m^−2^ s^−1^). This step is recommended to reactivate physiological activity and maintain the integrity of cell membranes (Honegger 2003). The experimental design is presented in Fig. 1. A large amount of lichen material (podetia) required for the entire duration of the experiment was prepared. A total of 25 experimental groups were considered. On the appropriate days of the experiment, the required amount of lichen material was collected for particular analyses. The experimental groups represented various combination doses of ammonium nitrate (NH_4_NO_3_; “nitrogen” factor) and sodium chloride (NaCl; “salt” factor). The applied doses were higher than those found in the environment due to the nature of the experiment, which was assumed to be one-time and short-term exposure to acute stress. The 0 M solution was a control group and constituted distilled water. Lichen thalli were placed in the vessels with individual solutions and shaken for 2 h on a vibrating shaker (rotation speed 250 rpm; Vibramax 100, Heidolph). Then, the thalli were transferred to a climatic chamber (ca. 95% relative humidity). After 1 h, the chlorophyll fluorescence was measured in 6 replicates for each experimental group. Next, the thalli were left in the climatic chamber, and after 24 h and 72 h, the measurements were made again. After 72 h, the thalli were intended for assessment of cell membrane integrity.

### Integrity of cell membranes

After treatment with solutions, the thalli were gently rinsed in deionized water to remove unbounded ions (Yemets et al. 2015). Ca 100 mg of air-dried lichen material was weighed and intended for analysis. The initial electrical conductivity in μS cm^−1^ (*Ci*) of the distilled water was measured using a pH/conductivity meter (Seven Go Duo SG23-FK5, Mettler Toledo). The samples were placed in 50 ml of distilled water in glass weight bottles, sealed with glass stoppers, and shaken on a vibrating shaker for 1 h (Vibramax 100, Heidolph). The conductivity of the samples was measured after soaking the thalli (*Cv*). The thalli were then boiled for 10 min at 100 °C to disrupt the cell membranes. The conductivity after the samples had cooled down to 20 °C was measured again (*Cf*). Relative electrical conductivity (*EC*), indicating the loss of cell membrane integrity, was calculated according to the formula: ((*Cv*–*Ci*)/*Cf*) × 100 (%). Five replicates for each experimental group were included. See Osyczka and Rola (2019) for details on the measurement procedure.

### Chlorophyll fluorescence measurements

The chlorophyll fluorescence measurements were conducted using a Handy-PEA + fluorimeter (Plant Efficiency Analyzer, Hansatech Instruments Ltd, UK). The 20 min of dark adaptation was applied before taking measurements. Chlorophyll fluorescence was induced by red light (wavelength 650 nm) provided by high-intensity LEDs. The saturating light pulse (2400 µmol/m^2^/s) was applied. All the fluorescence transients were recorded with a time span from 10 μs to 1 s.

The photosynthetic efficiency of the lichen photobiont was determined by the maximum PSII quantum yield arising from *chl a* fluorescence emission: *F*_*V*_*/F*_*M*_ = (*F*_*M*_ – *F*_*0*_)/*F*_*M*_, where *F*_*0*_ is the minimum and *F*_*M*_ maximum *chl a* fluorescence; whereas *F*_*V*_ = (*F*_*M*_ – *F*_*0*_) constituted variable fluorescence. Other chlorophyll fluorescence parameters were analyzed for a more precise analysis of photosynthetic apparatus condition (see Table S1).

### Statistical analysis

The significance of differences in *EC* and *F*_*V*_*/F*_*M*_ parameters in the control group between the thalli from polluted and unpolluted sites was verified by the Student’s *t*-tests (*p* < 0.05). Two-way analyses of variance (two-way ANOVA; *p* < 0.05) followed by Tukey’s HSD post hoc tests were performed to assess the effect of “salt” and “nitrogen” factors on the *EC* parameter, separately for lichen thalli collected from the polluted and unpolluted site. The proportions between *EC* of individual experimental groups with a certain salt dose without ammonium nitrate and with the highest concentration of ammonium nitrate (1 M) were calculated. Next, Student’s *t*-tests (*p* < 0.05) were used to verify the significance of differences between thalli collected from the polluted and unpolluted site in terms of the proportion of *EC* after treatment with solutions of the highest concentration of ammonium nitrate and without ammonium nitrate; separately for each concentration of the salt. Two-way analyses of variance (two-way ANOVA; *p* < 0.05) followed by Tukey’s HSD post hoc tests were applied to test the effect of “salt” and “nitrogen” factors on the *F*_*V*_*/F*_*M*_ parameter; separately for the thalli from the unpolluted and polluted site and for three-time intervals. Before the analyses, the normality distribution was checked with the Kolmogorov–Smirnov test, and the assumption of variance homogeneity in groups was checked using Levene’s test. The Box-Cox transformation was used where necessary. Statistical calculations were conducted in STATISTICA 12 (TIBCO Software Inc.).

The spider plots were created to visualise the influence of “salt” and “nitrogen” factors on the parameters characterising PSII functionality after 72 h in *Cladonia rei* thalli collected from the unpolluted and polluted site. Four combinations of NaCl and NH_4_NO_3_ treatment and a control group were included. Graphs were used to check which parameters responded most strongly to the effect of salt and nitrogen stress. The plots were based on the values normalised to the control treatment, which made it possible to compare the parameters measured on the various scales.

## Results

### Loss of cell membrane integrity

**Fig. 2:**
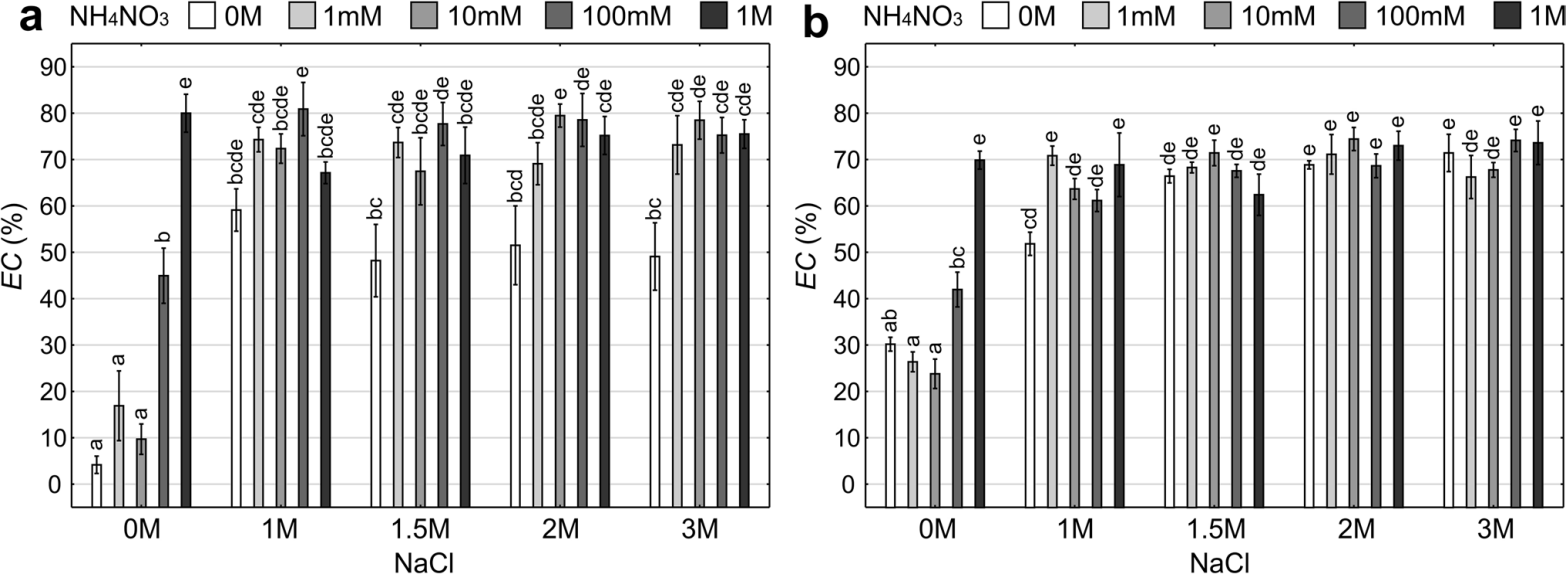
Loss of cell membrane integrity expressed with the *EC* in particular experimental groups (means ± SE; *n* = 5) for *Cladonia rei* collected from the unpolluted site (**a**) and polluted site (**b**). The different letters above the bars indicate statistically significant differences (*p* < 0.05). See Table S2 for details on the main effects and interactions

**Fig. 3:**
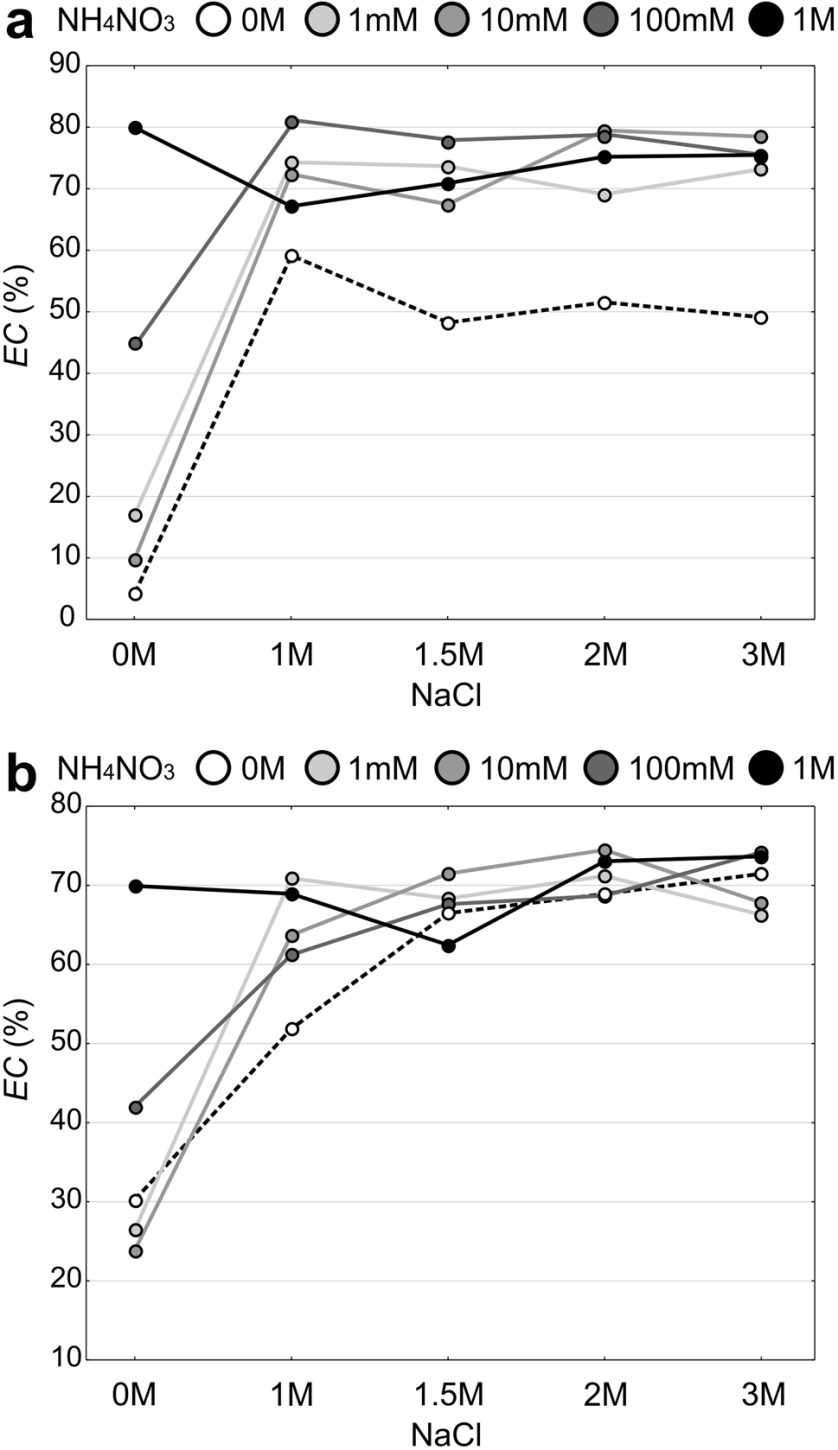
The relationship between the “salt” and “nitrogen” factors and the level of cell membrane damage (*EC*) presenting the interaction effect of the analysed factors for *Cladonia rei* collected from the unpolluted site (**a**) and polluted site (**b**)

In the case of lichen thalli collected from the unpolluted and polluted site, significant interactions between “salt” and “nitrogen” factors have been recorded (Fig. 2a, b, Fig. 3, Table S2). The lowest *EC* values were recorded for control and 0 M salt solution in combination with the lowest concentrations of ammonium nitrate solutions (1 mM, 10 mM, 100 mM) in the thalli collected from both habitats. However, the cell membrane damage in the control group from the polluted site was significantly higher compared to the unpolluted site (Student’s *t*-test; *p* < 0.05). As regards the unpolluted site, the salt treatment free of ammonium nitrate caused a lower loss of cell membrane integrity compared to the salt and ammonium nitrate combination. As a rule, in the case of simultaneous effect of NaCl and NH_4_NO_3_, *EC* increased along with increasing concentrations of ammonium nitrate (Fig. 2a). With regard to the polluted habitat, *EC* reached a similar level in the case of 1.5 M, 2 M, and 3 M salt solutions, both without and in combination with ammonium nitrate (Figs. 2b and 3). The highest values of *EC* were recorded in samples treated with salt solutions with the addition of ammonium nitrate in the highest concentrations: 2 M NaCl × 1 M NH_4_NO_3_, 3 M × 100 mM, and 3 M × 1 M, respectively (Fig. 2b). After treatment with different doses of salt, differences between the groups without and with the addition of ammonium nitrate were the most evident for all salt treatments in the case of thalli from the unpolluted site, and only for the lowest salt concentration in the case of thalli from the polluted site (Fig. 3).

**Fig. 4:**
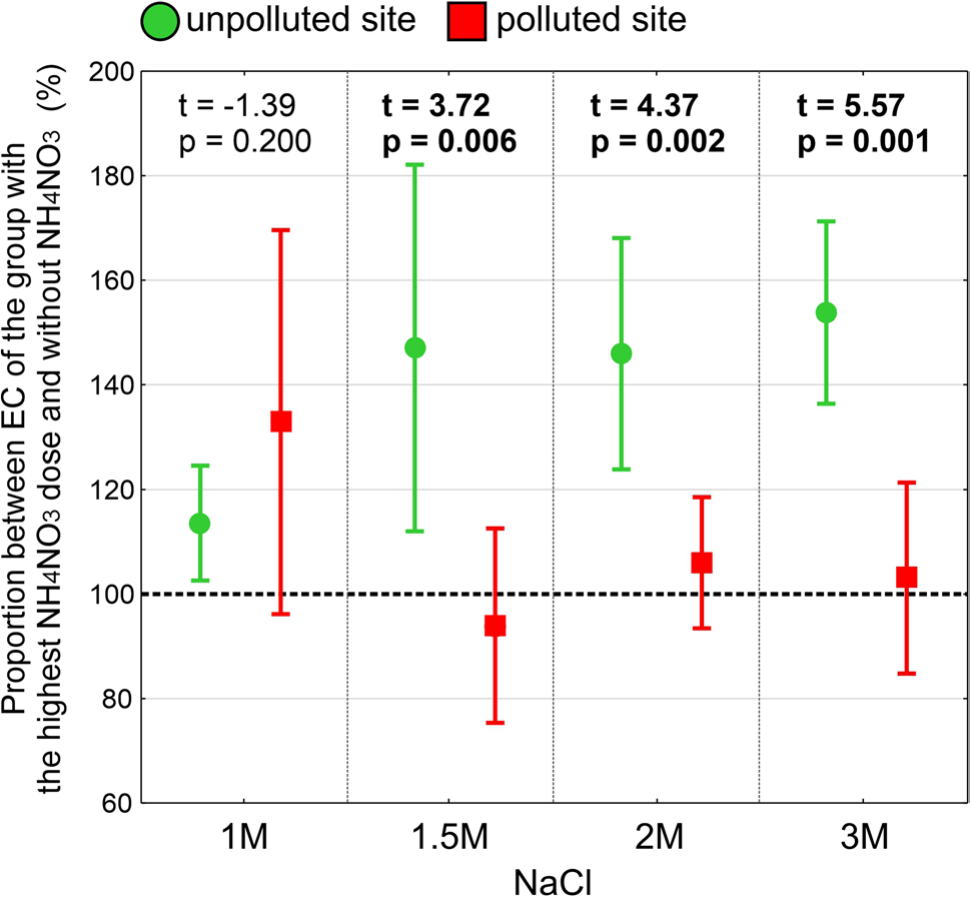
The proportion between *EC* of the experimental group with a certain salt dose (visible on axis X) with the highest NH_4_NO_3_ dose and the group of the same salt dose without NH_4_NO_3_ for *Cladonia rei* collected from the unpolluted site and polluted site. The dotted line at 100% indicates that for a particular salt solution concentration, *EC* was the same both in the absence and presence of NH_4_NO_3_. The results of the Student’s *t*-test (*p* < 0.05) verifying the significance of differences between the lichen thalli collected from two different sites within the same salt solution concentration are provided

The comparison of the proportion between *EC* of the experimental group with a certain salt dose with addition of the highest NH_4_NO_3_ dose and without NH_4_NO_3_ revealed a significant difference in the response of lichens collected from the unpolluted and polluted habitat (Fig. 4). In the thalli collected from the unpolluted habitat, a much stronger negative effect of ammonium nitrate was noted in comparison with the thalli collected from the polluted site (higher proportion between *EC* for the thalli treated with salt with addition of the highest NH_4_NO_3_ dose in relation to those treated with salt alone). In the case of thalli from the polluted site, *EC* values were similar after treatment with 1.5 M salt solutions and higher, both in the absence and presence of ammonium nitrate. On the other hand, in the case of thalli collected from an unpolluted habitat, in each dose of salt used, significantly higher *EC* values were recorded in samples treated simultaneously with ammonium nitrate than in the case of its absence. Moreover, the differences between the solutions with and without ammonium nitrate increased along with the increasing concentration of the salt solution. Significant differences in the response of thalli collected from the unpolluted and polluted site were recorded for 1.5 M, 2 M, and 3 M NaCl solutions (Fig. 4).

### Photosynthetic efficiency

**Fig. 5:**
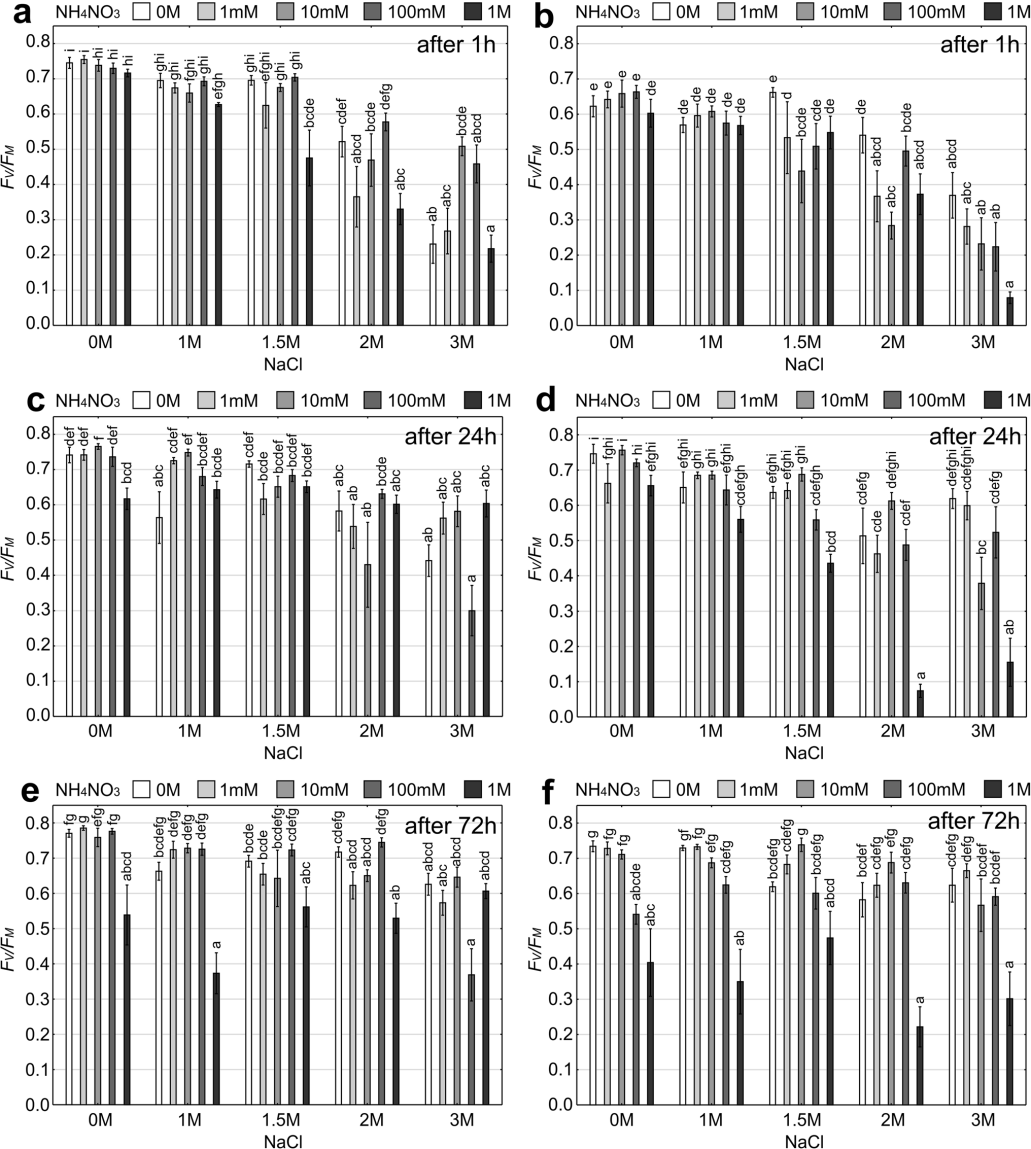
*F*_*V*_*/F*_*M*_ parameter in particular experimental groups (means ± SE; *n* = 6) representing different combinations of ammonium nitrate and sodium chloride solutions after 1 h, 24 h and 72 h for *Cladonia rei* collected from unpolluted habitat (**a, c, e**) and polluted habitat (**b, d, f**). The different letters above the bars indicate statistically significant differences (*p* < 0.05). See Table S3 for details on the main effects and interactions

Comparing the *F*_*V*_*/F*_*M*_ in the control groups from both sites, the thalli from the polluted site were characterized by significantly lower *F*_*V*_*/F*_*M*_ values immediately after exposure to stress compared to the thalli from the unpolluted site (Student’s *t*-test; *p* < 0.05). The maximum quantum yield of PSII photochemistry was influenced by both “nitrogen” and “salt” factors in unpolluted and polluted habitats for each measurement time (Fig. 5, Table S3). In the case of thalli from an unpolluted site, after 1 h from the treatment, significant differences in *F*_*V*_*/F*_*M*_ were observed between the solution of salt alone and 1.5 M and 2 M salt solution with the addition of the highest concentration of ammonium nitrate. The lowest *F*_*V*_*/F*_*M*_ value was recorded for treatment with a combination of the highest concentrations of salt and ammonium nitrate solutions; this group differed significantly from most experimental groups (Fig. 5a). After 24 h and 72 h, *F*_*V*_*/F*_*M*_ increased in most experimental groups compared to the measurement after 1 h (Fig. 5c, e). As regards the polluted site, after 1 h of treatment visible decrease in the *F*_*V*_*/F*_*M*_ parameter was noted for 2 M and 3 M NaCl, especially in combinations with ammonium nitrate (Fig. 5b). The lowest value of *F*_*V*_*/F*_*M*_ was observed after treatment with 3 M NaCl and 1 M NH_4_NO_3_. After 24 h, *F*_*V*_*/F*_*M*_ increased in most of the experimental groups, except for the combinations of 2 M and 3 M salt solution with the addition of 1 M ammonium nitrate (Fig. 5d). The situation after 72 h was very similar; however, *F*_*V*_*/F*_*M*_ decreased in the experimental groups which consisted of combinations of low salt concentrations and 1 M ammonium nitrate (Fig. 5f).

**Fig. 6:**
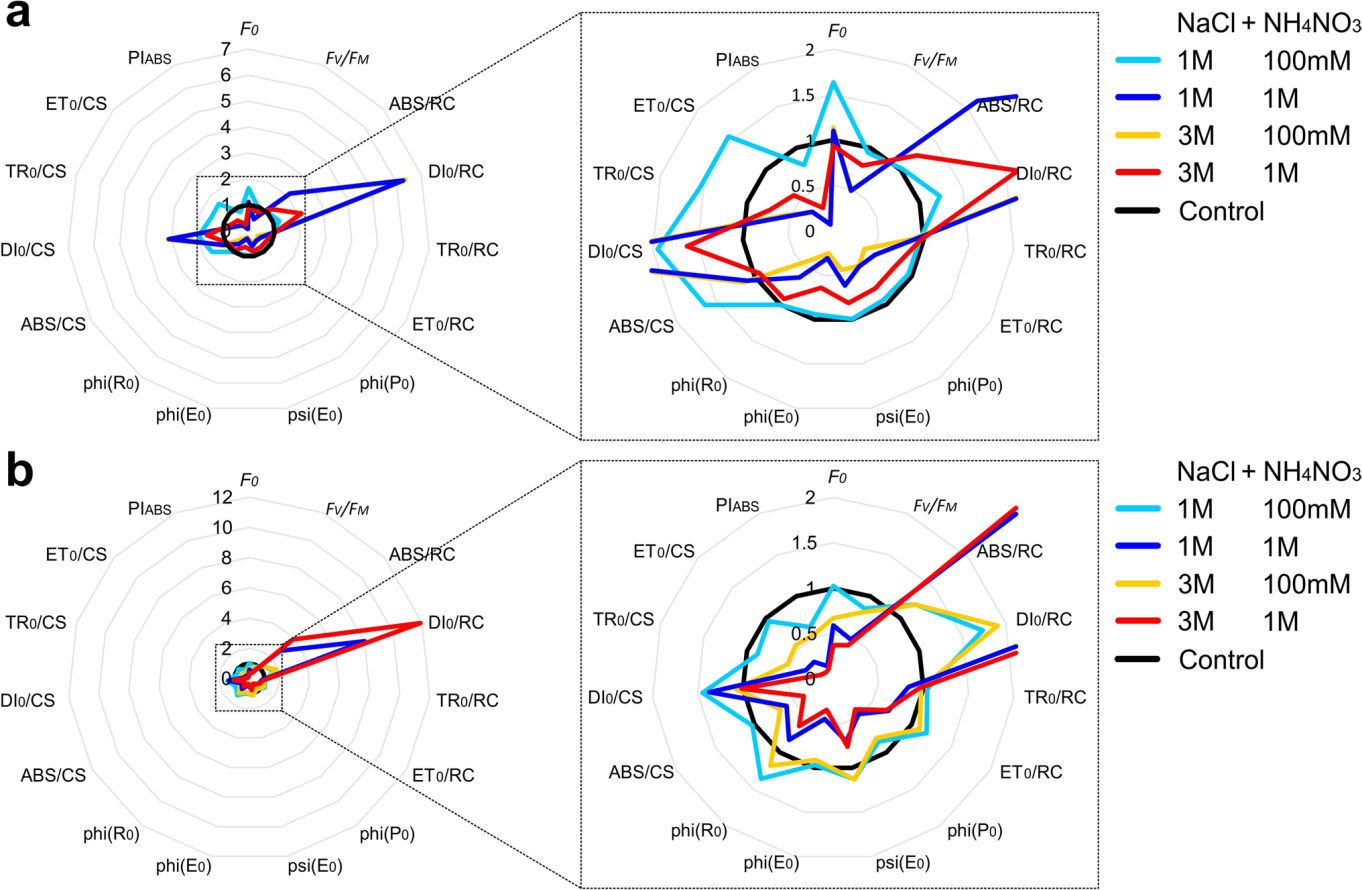
The spider plots showing the effect of different combinations of NaCl and NH_4_NO_3_ treatments on various photosynthetic parameters characterising PSII functionality after 72 h in *Cladonia rei* lichen thalli collected from the unpolluted (**a**) and polluted (**b**) site. The plots are based on normalized values to control treatment, enabling comparison of the variables measured on different scales. A detailed description of all parameters is provided in Table S1

A comparison of the chlorophyll fluorescence parameters for the experimental groups in 4 combinations of the lowest and highest salt and ammonium nitrate concentrations revealed differences in these parameters in relation to the control (Fig. 6, Table S1). Thalli from the unpolluted site were characterized by higher values of DI_0_/RC, DI_0_/CS, and lower values of PI_ABS_ in all analyzed combinations in relation to the control. The highest values of DI_0_/RC, DI_0_/CS and lowest of PI_ABS_ were recorded for combinations with the highest salt and ammonium nitrate concentrations (Fig. 6a). Besides the combination of the lowest salt concentration and the lowest ammonium nitrate concentration, the thalli representing all other experimental groups had lower values of phi(R_0_), phi(E_0_), psi(E_0_), phi(P_0_), and ET_0_/RC relative to the control. As regards thalli from polluted habitats, a marked increase was observed in DI_0_/RC and ABS/RC parameters compared to the control, especially in combinations of salt and ammonium nitrate of the highest concentrations. The greatest decreases in *F*_*V*_*/F*_*M*_, *F*_*0*_, PI_ABS_, phi(R_0_), phi(E_0_), psi(E_0_), phi(P_0_), ET_0_/RC, and ET_0_/CS were also observed in the same experimental groups (Fig. 6b).

## Discussion

### The effect of combined salt and nitrogen stress on cell membrane integrity

Electrical conductivity measurement is a well-known method to test responses of lichens exposed to various kinds of environmental stress, e.g., SO_2_, nitrogen excess, heavy metals, and light exposure (e.g., Garty et al. 1998; Marques et al. 2005; Munzi et al. 2009; Yemets et al. 2015; Osyczka and Rola 2019). This parameter is mainly a mycobiont stress indicator because the fungal partner constitutes approx. 90% of total lichen biomass (Munzi et al. 2009; Gasulla et al. 2021). We found that high concentrations of ammonium nitrate increased cell membrane damage; however, in combination with salt, the level of cell membrane integrity was frequently even lower. Thus, the simultaneous effect of NaCl and NH_4_NO_3_ has the most detrimental effect. Munzi et al. (2009) found that non-nitrophilous *Evernia prunastri* showed a decrease of cell membrane integrity after rinsing with KNO_3_, NH_4_NO_3_, and (NH_4_)_2_SO_4_ solutions. Nitrogen excess can directly influence either one symbiotic partner or the whole thallus through the changes in nutrient uptake and balance between bionts. However, it depends on the reaction of the species in question and dose of supplied nitrogen. For example, Munzi et al. (2010) observed that, in *Evernia prunastri* exposed to ammonium loads corresponding to real environmental conditions, no membrane damage occurred after 5 weeks of exposure. Considering these results and ours, it can be concluded that, in the case of non-nitrophilous lichens, acute nitrogen stress seems to result in more severe damage to cells than chronic, longer exposure to low doses of nitrogen.

We found that samples exposed to environmental stress prior to the experiment had a higher level of cell membrane damage in the control treatment than the thalli collected from the unpolluted habitat. This suggests that other stress factors like heavy-metal pollution may impair the condition of lichens. Similarly, Osyczka and Rola (2019) found that lichens inhabiting highly polluted areas showed greater cell membrane damage than those growing in low polluted sites. Interestingly, lichen individuals exposed to environmental stress before the experiment did not respond significantly stronger to a combination of salt and ammonium nitrate treatment than lichen thalli collected from an unpolluted habitat. The only symptomatic difference between the thalli collected from two different sites was a stronger negative reaction to pure salt solutions of high concentrations in lichens that originated from a polluted site. This suggests that salt stress can cause more negative consequences in lichens exposed to other kinds of environmental stress.

Finally, both high doses of salt alone, ammonium nitrate alone, and the combination thereof result in a severe disturbance of the integrity of cell membranes compared to the control samples. This confirms that the mycobiont is sensitive to these factors and the level of cell membrane damage can serve as a relevant indicator of acute combined salt-nitrogen stress incidents as also previously found in the reactions of lichens exposed to stress caused by heavy metals or various forms of nitrogen (Munzi et al. 2009; Osyczka and Rola 2019).

### Photosynthetic efficiency response to nitrogen and salt stress

The measurements of chlorophyll fluorescence are widely used to study the effect of physiological stress induced by numerous factors on photosynthesis. This technique has been used to investigate the effect of salt stress on different lichen species (e.g., Nash and Lange 1988; Matos et al. 2011; Malaspina et al. 2015, Gasulla et al. 2019, Chowaniec et al. 2022). The effect of various forms of nitrogen on lichen photosynthesis has also been the subject of several studies (e.g., Paoli et al. 2010; Munzi et al. 2012; Maslaňáková et al. 2015). However, the interaction of these two factors on lichen physiology has not been studied thus far.

In our study, the maximum quantum yield of PSII decreased particularly after treatment with a combination of the highest concentrations of salt and ammonium nitrate solutions. Several studies showed that non-nitrophilous species proved to be particularly sensitive to high doses of nitrogen. For example, Paoli et al. (2010) observed decreased *F*_*V*_*/F*_*M*_ in *Evernia prunastr*i and *Pseudevernia furfuracea* exposed to atmospheric ammonia (NH_3_). Morillas et al. (2021) tested the combined effect of (NH_4_)_2_SO_4_ and solar radiation on six epiphytic lichen species and noticed a decrease in *F*_*V*_*/F*_*M*_. The study on the effect of ammonium chloride on *E. prunastri* also showed a reduction of *F*_*V*_*/F*_*M*_ due to stress (Munzi et al. 2012). The effect of ammonium nitrate on representatives of *Cladonia* lichens was tested by Maslaňáková et al. (2015). They observed significant differences of *F*_*V*_*/F*_*M*_ in *C. arbuscula* subsp. *mitis* only after 24 h, but not after 48 h (Maslaňáková et al. 2015). This may be explained by the fact that lichens adapted or recovered. However, the response to nitrogen excess turns out to be species specific. For example, tolerance to N supplied in excess of nitrophilous *Xanthoria parietina* results from low cation exchange capacity that limits nitrogen binding to cell walls, thereby enhancing resistance to the harmful impact of nitrogen (Gaio-Oliveira et al. 2001, 2005). Our results showed that short-term stress with small doses of nitrogen (up to 100 mM NH_4_NO_3_) did not cause significant changes in the *F*_*V*_*/F*_*M*_ parameter compared to the control. On the other hand, the 1 M solution caused a significant decrease in this parameter only after 24 h; an even greater decrease was observed after 72 h. This result is consistent with the fact that *Cladonia rei* is a species intolerant to environmental eutrophication (Nimis 2016).

With regard to salt impact, the exposure to short-term acute stress decreased the photosynthetic efficiency in lichens. Nevertheless, after 72 h, the photosynthesis efficiency level returned to the level characteristic of healthy lichens in experimental groups without and with small doses of ammonium nitrate. Similarly, Matos et al. (2011) found that the maximum quantum yield of PSII decreased in *Ramalina canariensis* after incubation in 20% artificial seawater and observed the recovery of *F*_*V*_*/F*_*M*_ to control levels after 48 h; however, regeneration was not observed at higher concentrations. Our results indicate that short-term salt stress causes only a temporary reduction in photosynthesis efficiency, and in a nitrogen-poor environment, the photosynthesis efficiency may regenerate over time. Such a result suggests that the effects of salt stress are related mainly to the osmotic effect and that after rehydration, the photobiont could regain its performance, in contrast to the mycobiont, in which we observed a high level of cell membrane damage after 72 h. Therefore, it can be concluded that in the case of *C. rei,* a single episode of exposure to a large dose of salt does not result in irreversible damage to photosynthesis but only temporarily reduces photosynthetic efficiency, which improves over time. Lichens are poikilohydric organisms and are often exposed to episodes of dehydration, which is also associated with osmotic stress. They can survive such stress without physiological damage (Honegger et al. 1996). Apart from osmotic stress, excessive salinity can lead to more damaging ionic stress; therefore, the observed reversibility of the changes suggests that the lichens were exposed to salt stress for too short a time for strong ionic stress to trigger irreversible changes in photosynthesis. However, in combination with high nitrogen doses, recovery of photosynthetic efficiency to a level corresponding to healthy lichens was not possible. This means that in urban environments where lichens are exposed to high doses of nitrogen, the additional exposure to salt may have much more severe consequences for the condition of these organisms. Mechanisms of toxicity of nitrogen compound on lichens may result not only from increased N levels in the thallus but also from a possible change of the balanced exploitation of N between photobiont and mycobiont if one of them is favored over the other due to altered nutrient supply (Turpin 1991; Gries 1996). Finally, one should remember that lichen species can vary significantly in response to salinity stress. For example, Nash and Lange (1988) observed in *Pseudocyphellaria anomala* decrease in net photosynthesis after exposure to 100% sea-salt solution. This effect was partially reversible only if the exposure lasted less than 10 h. A similar response was observed in epiphytic lichens by Chowaniec and Rola (2022), who found that *Hypogymnia physodes* was more sensitive to salt stress than *Pseudevernia furfuracea*; however, the reduction of *F*_*V*_*/F*_*M*_ was not permanent since the values returned to the control level after 24 h. On the other hand, the photobiont *Halofilum ramosum*, isolated from lichens associated with seacoasts, showed no clear reduction in *F*_*V*_*/F*_*M*_ in relation to NaCl concentration (Gasulla et al. 2019).

*Cladonia rei* collected from a polluted site had lower *F*_*V*_*/F*_*M*_ values after exposure to combined salt-nitrogen stress compared to the lichen thalli from an unpolluted site. Exposure to high levels of heavy metals may have caused lichens collected from the polluted site to develop various defence mechanisms to inactivate the reactive oxygen species (ROS) generated by stress. This may indicate that although the initial photosynthetic efficiency of lichens from both sites was similar, only exposure to a different type of stress reveals their more robust response to stress factors and the associated greater physiological damage. This is particularly important for lichens inhabiting the vicinity of roads in cities because, apart from exposure to salt and nitrogen stress, they are also under the influence of the increased concentrations of heavy metals emitted by road traffic. Contrarily, Munzi et al. (2011) found that *Xanthoria parietina* samples from an N-enriched site had a higher N-stress recovery capacity than samples from an area with low nitrogen emissions. This indicates that exposure to nitrogen stress in the native environment gives this species a better ability to deal with the effects of nitrogen pollution, and tolerance to nitrogen excess is not only a species-specific attribute but also a feature induced by the environment in which a lichen grows (Munzi et al. 2011).

### Chlorophyll fluorescence parameters

We observed the most significant increase of DI_0_/RC and ABS/RC parameters in relation to the control for combinations with the highest salt and ammonium nitrate concentrations. The DI_0_/RC value indicates how much energy which achieves the PSII reaction centres is dispersed mainly in the form of heat (Strasser et al. 2000). In our case, the combination of high salt with a low dose of nitrogen did not cause such a significant increase in DI_0_/RC as in the case of a high dose of salt combined with the highest doses of nitrogen. Moreover, the highest levels of DI_0_/RC were recorded at high doses of nitrogen, regardless of whether the salt concentration was the highest or the lowest. This may suggest that the high doses of nitrogen enhanced dissipation energy flux per reaction centre to the greatest extent. Similarly, Paoli et al. (2010) tested the impact of NH_3_ on *P. furfuracea* and *Evernia prunastri* and observed a considerably elevated level of DI_0_/RC.

The increase in specific absorption flux per reaction centre (ABS/RC) can result from the inactivation of some PS II reaction centres or a rise in antenna size (Kalaji et al. 2016); however, changes in this parameter depend on the type of stress and species. Increased ABS/RC with TR_0_/RC after desiccation of thalli indicate that the number of active RCs is reduced by the dehydration process in lichens (Bednaříková et al. 2020). However, the response of various species to stress may differ, as shown in the example of *Usnea antarctica* and *U. aurantiaco-atra* where, after photoinhibition treatment, ABS/RC and TR_0_/RC increased in the first species but decreased in the second (Balarinová et al. 2014). Moreover, the observed in our study increase in values of DI_0_/CS in all combinations of salt and nitrogen treatments compared to the control could indicate that the reaction centres trigger defence mechanisms in response to stress (Wang et al. 2019b).

We observed clear differences between the lichens collected from both sites in the case of minimal fluorescence (*F*_*0*_). The parameter had a similar value to the control in lichens collected from the unpolluted area, while a decrease of *F*_*0*_ was noticeable in most experimental groups in lichens from the polluted site. This may indicate a more pronounced reaction of lichens exposed to stress in their native environment. The decrease in *F*_*0*_ may indicate non-photochemical energy dissipation by photosystem II antenna pigments (Li et al. 2013). Different types of stress factors trigger this phenomenon in lichens. For example, a decrease in *F*_*0*_ was caused by light stress (Balarinová et al. 2014), salt and sucrose stress (Jensen et al. 1999), desiccation (Bednaříková et al. 2020) and biocides (Vannini et al. 2018).

The combination of high doses of salt and ammonium nitrate also resulted in a decrease in ET_0_/RC and ET_0_/CS. Jajoo (2013) also reported a decrease in the electron transport per excited cross section due to the inactivation of the reaction centre complex after treating plants with various NaCl concentrations. Our results indicated that the PI_ABS_ parameter was more sensitive to the combination of salt and ammonium nitrate than *F*_*V*_*/F*_*M*_. Other studies showed similar results in lichens exposed to NH_3_ (Paoli et al. 2010) or NH_4_Cl (Munzi et al. 2012). Furthermore, Jafarinia and Shariati (2012) found PI_ABS_ as a useful photosynthetic parameter to test sensitivity to salinity stress in *Brassica napus*, which was also confirmed in the case of lichens by Chowaniec and Rola (2022).

## Conclusions

Exposure to a combination of sodium chloride and ammonium nitrate, especially in high doses, cause significant disturbances in the physiology of *C. rei*. Therefore, when planning research on the impact of stress factors occurring in the urban environment on lichens, it is crucial to consider the combinations of various factors that interact and occur in high concentrations in a given habitat simultaneously.

The combined effect of NaCl and NH_4_NO_3_ has the most detrimental effect on the integrity of cell membranes in the studied lichen. Both high doses of salt alone, ammonium nitrate alone, and the combination thereof result in a severe disturbance of the integrity of cell membranes compared to the control samples. This confirms that mycobiont is sensitive to these factors and that the level of cell membrane damage can be a relevant indicator of acute combined salt-nitrogen stress incidents.

The exposure of the studied lichen species to short-term salt stress only causes a temporary reduction in photosynthesis efficiency; however, in a nitrogen-poor environment, the photosynthesis efficiency may regenerate over time. Nevertheless, in urban environments where lichens are exposed to high doses of nitrogen, additional exposure to salt may have much more serious consequences for the condition of these organisms.

Lichens in a habitat where other environmental stress was present responded more strongly to short-term combined salt-nitrogen stress than those from a habitat without significant stress factors. This indicates that the physiological condition of lichens previously affected by certain harmful factors may be weakened, and exposure to another type of stress factor may lead to greater physiological damage to both photobiont and mycobiont. This is particularly important for lichens inhabiting the vicinity of roads because, apart from exposure to salt and nitrogen stress, they are also influenced by increased concentrations of pollutants emitted by road traffic.

## Supplementary Information

Below is the link to the electronic supplementary material.

Supplementary file1 (PDF 120 KB)

## Data Availability

The datasets used and analyzed during the current study are available from the corresponding author on request.

## References

[CR1] Ahmadjian V (1993). The lichen symbiosis.

[CR2] Baker LA, Brazel AT, Westerhoff P (2004). Environmental consequences of rapid urbanization in warm, arid lands: Case study of Phoenix, Arizona (USA). Adv Archit Ser.

[CR3] Balarinová K, Barták M, Hazdrová J, Hájek J, Jílková J (2014). Changes in photosynthesis, pigment composition and glutathione contents in two Antarctic lichens during a light stress and recovery. Photosynthetica.

[CR4] Bednaříková M, Váczi P, Lazár D, Barták M (2020). Photosynthetic performance of Antarctic lichen *Dermatocarpon polyphyllizum* when affected by desiccation and low temperatures. Photosynth Res.

[CR5] Chowaniec K, Rola K (2022). Evaluation of the importance of ionic and osmotic components of salt stress on the photosynthetic efficiency of epiphytic lichens. Physiol Mol Biol Plants.

[CR6] Chowaniec K, Latkowska E, Rola K (2022). Does long-term salt stress of environmentally relevant concentrations affect the physiology of inland lichens? – The importance of rainfall to restore thallus vitality. Environ Exp Bot.

[CR7] Cohen AJ, Anderson HR, Ostro B, Pandey KD, Krzyzanowski M, Künzli N, Gutschmidt K, Arden Pope III, C, Romieu I, Samet JM, Smith KR, , Ezzati M, Lopez AD, Rodgers A, Murray CJL (2004). Urban air pollution. Comparative Quantification of Health Risks: Global and Regional Burden of Disease Attribution to Selected Major Risk Factors.

[CR8] Cunningham M, Snyder E, Yonkin D, Ross M, Elsen T (2008). Accumulation of deicing salts in soils in an urban environment. Urban Ecosyst.

[CR9] Dahlman L, Persson J, Pamlqvist K, Näsholm T (2004). Organic and inorganic nitrogen uptake in lichens. Planta.

[CR10] Decina SM, Hutyra LR, Templer PH (2019). Hotspots of nitrogen deposition in the world's urban areas: a global data synthesis. Front Ecol Environ.

[CR11] Decina SM, Templer PH, Hutyra LR, Gately CK, Rao P (2017). Variability, drivers, and effects of atmospheric nitrogen inputs across an urban area: Emerging patterns among human activities, the atmosphere, and soils. Sci Total Environ.

[CR12] Delmail D, Grube M, Parrot D, Cook-Moreau J, Boustie J, Labrousse P, Tomasi S, Prasad MNV, Ahmad P, Azooz MM (2013). Halotolerance. In: lichens: symbiotic coalition against salt stress. Ecophysiology and Responses of Plants under Salt Stress.

[CR13] Engardt M, Simpson D, Schwikowski M, Granat L (2017) Deposition of sulphur and nitrogen in Europe 1900–2050. Model calculations and comparison to historical observations. Tellus B 69:1328945. 10.1080/16000889.2017.1328945

[CR14] Erdmann N, Hagemann M (2001) Salt acclimation of algae and cyanobacteria: a comparison. In: Rai LC, Gaur JP (eds) Algal adaptation to environmental stresses: physiological, biochemical and molecular mechanisms, 1st ed. Berlin, Heidelberg, pp. 323–361

[CR15] Erisman JW, Bleeker A, Galloway J, Sutton MS (2007). Reduced nitrogen in ecology and the environment. Environ Pollut.

[CR16] Frati L, Brunialti G, Loppi S (2008). Effects of reduced nitrogen compounds on epiphytic lichen communities in Mediterranean Italy. Sci Total Environ.

[CR17] Frati L, Caprasecca E, Santoni S, Gaggi C, Guttova A, Gaudino S, Pati A, Rosamilia S, Pirintsos SA, Loppi S (2006). Effects of NO_2_ and NH_3_ from road traffic on epiphytic lichens. Environ Pollut.

[CR18] Gaio-Oliveira G, Dahlman L, Palmqvist K, Máguas C (2004). Ammonium uptake in the nitrophytic lichen *Xanthoria parietina* and its effects on vitality and balance between symbionts. Lichenologist.

[CR19] Gaio-Oliveira G, Branquinho C, Máguas C, Martins-Loução MA (2001). The concentration of nitrogen in nitrophilous and non-nitrophilous lichen species. Symbiosis.

[CR20] Gaio‐Oliveira G, Dahlman L, Palmqvist K, Martins‐Loução MA, Máguas C (2005) Nitrogen uptake in relation to excess supply and its effects on the lichens *Evernia prunastri* (L.) Ach and *Xanthoria parietina* (L.) Th Fr Planta 220:794–803. 10.1007/s00425-004-1396-1 10.1007/s00425-004-1396-1 15503128

[CR21] Galloway JN, Schlesinger WH, Levy H, Michaels A, Schnoor JL (1995). Nitrogen fixation: anthropogenic enhancement e environmental response. Glob Biochem Cycles.

[CR22] Garty J, Kloog N, Cohen Y (1998). Integrity of lichen cell membranes in relation to concentration of airborne elements. Arch Environ Contam Toxicol.

[CR23] Gasulla F, Guéra A, de los Ríos A, Pérez-Ortega S (2019). Differential responses to salt concentrations of lichen photobiont strains isolated from lichens occurring in different littoral zones. Plant Fungal Syst.

[CR24] Gasulla F, del Campo EM, Casano LM, Guéra A (2021). Advances in understanding of desiccation tolerance of lichens and lichen-forming algae. Plants.

[CR25] Godwin KS, Hafner SD, Buff MF (2003). Long-term trends in sodium and chloride in the Mohawk River, New York: the effect of fifty years of road-salt application. Environ Pollut.

[CR26] Gombert S, Asta J, Seaward MRD (2003). Correlation between the nitrogen concentration of two epiphytic lichens and the traffic density in an urban area. Environ Pollut.

[CR27] Gries C, Nash TH (1996). Lichens as indicators of air pollution. Lichen biology.

[CR28] Hasegawa PM, Bressan R, Zhu JK, Bohnert HJ (2000). Plant cellular and molecular responses to high salinity. Annu Rev Plant Physiol Plant Mol Biol.

[CR29] Honegger R, Peter M, Scherrer S (1996). Drought-induced structural alterations at the mycobiont-photobiont interface in a range of foliose macrolichens. Protoplasma.

[CR30] Honegger R (2003). The impact of different long-term storage conditions on the viability of lichen-forming ascomycetes and their green algal photobiont, *Trebouxia* spp. Plant Biol.

[CR31] Howard KWF, Haynes J (1993). Groundwater contamination due to road de-icing chemicals — salt balance implications. Geosci Can.

[CR32] Jafarinia M, Shariati M (2012). Effects of salt stress on photosystem II of canola plant (*Brassica napus* L.) probing by chlorophyll *a* fluorescence measurements. Iran J Sci Technol.

[CR33] Jajoo A (2013) Changes in photosystem II in response to salt stress. In: Ahmad P, Azooz M, Prasad M (eds) Ecophysiology and Responses of Plants under Salt Stress. Springer, New York, NY. 10.1007/978-1-4614-4747-4_5

[CR34] James PW, Smith CW, Aptroot A, Coppins BJ, Fletcher A, Gilbert OL, James PW, Wolseley PA (2009). *Cladonia* P. Browne (1756). The lichens of Great Britain and Ireland.

[CR35] Jamshidi A, Goodarzi AR, Razmara P (2020). Long-term impacts of road salt application on the groundwater contamination in urban environments. Environ Sci Pollut Res.

[CR36] Jensen M, Chakir S, Feige G (1999). Osmotic and atmospheric dehydration effects in the lichens *Hypogymnia physodes*, *Lobaria pulmonaria*, and *Peltigera aphthosa*: an in vivo study of the chlorophyll fluorescence induction. Photosynthetica.

[CR37] Kalaji HM, Jajoo A, Oukarroum A, Brestic M, Zivcak M, Samborska IA, Cetner MD, Łukasik I, Goltsev V, Ladle RL (2016). Chlorophyll *a* fluorescence as a tool to monitor physiological status of plants under abiotic stress conditions. Acta Physiol Plant.

[CR38] Laceby P, Kerr JG, Zhu D, Chung C, Situ Q, Abbasi S, Orwin JF (2019). Chloride inputs to the North Saskatchewan River watershed: the role of road salts as a potential driver of salinization downstream of North America's northern most major city (Edmonton, Canada). Sci Total Environ.

[CR39] Li GL, Wu HX, Sun YQ, Zhang SY (2013). Response of chlorophyll fluorescence parameters to drought stress in sugar beet seedlings. Russ J Plant Physiol.

[CR40] Ludwikowski JJ, Peterson EW (2018). Transport and fate of chloride from road salt within a mixed urban and agricultural watershed in Illinois (USA): assessing the influence of chloride application rates. Hydrogeol J.

[CR41] Malaspina P, Giordani P, Pastorino G, Modenesi P, Mariotti G, Mauro G (2015). Interaction of sea salt and atmospheric pollution alters the OJIP fluorescence transient in the lichen *Pseudevernia furfuracea* (L.) Zopf. Ecol Indic.

[CR42] Marques AP, Freitas MC, Wolterbeek HT, Steinebach OM, Verburg T, De Goeij JJ (2005). Cell-membrane damage and element leaching in transplanted *Parmelia sulcata* lichen related to ambient SO_2_, temperature, and precipitation. Environ Sci Technol.

[CR43] Maslaňáková I, Biľová I, Goga M, Kuchár M, Bačkor M (2015). Differences between sensitivity of mycobiont and photobiont of *Cladonia* sp. lichens to different types of nitrogen exposure. Water Air Soil Pollut.

[CR44] Matos P, Cardoso-Vilhena J, Figueira R, Sousa AJ (2011). Effects of salinity stress on cellular location of elements and photosynthesis in *Ramalina canariensis* Steiner. Lichenologist.

[CR45] Mishler BD, Oliver MJ (2018) Putting physcomitrella patens on the tree of life: the evolution and ecology of mosses. In: Knight CD, Perroud P-F, Cove. DJ (eds) Annual Plant Reviews; Wiley: Hoboken, NJ, USA. 10.1002/9781119312994.apr0384

[CR46] Miyamoto S, Chacon A (2006). Soil salinity of urban turf areas irrigated with saline water: II. Soil Factors Landsc Urban Plan.

[CR47] Morillas L, Roales J, Cruz C, Munzi S (2022). Non-toxic increases in nitrogen availability can improve the ability of the soil lichen *Cladonia rangiferina* to cope with environmental changes. J Fungi.

[CR48] Morillas L, Roales J, Cruz C, Munzi S (2021). Resilience of epiphytic lichens to combined effects of increasing nitrogen and solar radiation. J Fungi.

[CR49] Munzi S, Loppi S, Cruz C, Branquinho C (2011). Do lichens have “memory” of their native N environment?. Planta.

[CR50] Munzi S, Paoli L, Fiorini E, Loppi S (2012). Physiological response of the epiphytic lichen *Evernia prunastri* (L.) Ach. to ecologically relevant nitrogen concentrations. Environ Pollut.

[CR51] Munzi S, Pisani T, Loppi S (2009). The integrity of lichen cell membrane as a suitable parameter for monitoring biological effects of acute nitrogen pollution. Ecotoxicol Environ Saf.

[CR52] Munzi S, Pisani T, Paoli L, Loppi S (2010). Time- and dose-dependency of the effects of nitrogen pollution on lichens. Ecotoxicol Environ Saf.

[CR53] Nash TH, Lange OL (1988). Responses of lichens to salinity: concentration and time-course relationships and variability among Californian species. New Phytol.

[CR54] Nimis PL, Purvis OW (2002) Monitoring lichens as indicators of pollution. In: Nimis PL Scheidegger C, Wolseley PA (eds) Monitoring with lichens – Monitoring lichens. Kluwer Academic Publishers, Dordrecht, Boston, London, pp 7–10

[CR55] Nimis PL (2016) ITALIC - The Information System on Italian Lichens. Version 5.0. University of Trieste, Dept of Biology. http://dryades.units.it/italic. Accessed 5 Jan 2022

[CR56] Novotny EV, Murphy D, Stefan HG (2008). Increase of urban lake salinity by road deicing salt. Sci Total Environ.

[CR57] Nybakken L, Johansson O, Palmqvist K (2009). Defensive compound concentration in boreal lichens in response to simulated nitrogen deposition. Glob Change Biol.

[CR58] Osyczka P, Lenart-Boroń A, Boroń P, Rola K (2021). Lichen-forming fungi in postindustrial habitats involve alternative photobionts. Mycologia.

[CR59] Osyczka P, Rola K (2019). Integrity of lichen cell membranes as an indicator of heavy-metal pollution levels in soil. Ecotoxicol Environ Saf.

[CR60] Osyczka P, Rola K (2013). *Cladonia* lichens as the most effective and essential pioneers in strongly contaminated slag dumps. Cent Eur J Biol.

[CR61] Osyczka P, Rola K, Lenart-Boroń A, Boroń P (2014). High intraspecific genetic and morphological variation in the pioneer lichen *Cladonia rei* colonising slag dumps. Cent Eur J Biol.

[CR62] Paoli L, Munzi S, Fiorini E, Gaggi C, Loppi S (2013). Influence of angular exposure and proximity to vehicular traffic on the diversity of epiphytic lichens and the bioaccumulation of traffic-related elements. Environ Sci Pollut Res.

[CR63] Paoli L, Maslaňáková I, Grassi A, Bačkor M, Loppi S (2015). Effects of acute NH_3_ air pollution on N-sensitive and N-tolerant lichen species. Ecotoxicol Environ Saf.

[CR64] Paoli L, Pirintsos SA, Kotzabasis K, Pisani T, Navakoudis E, Loppi S (2010). Effects of ammonia from livestock farming on lichen photosynthesis. Environ Pollut.

[CR65] Pataki DE, Alig RJ, Fung AS, Golubiewski NE, Kennedy CA, McPherson EG, Nowak DJ, Pouyat RV, Romero Lankao P (2006). Urban ecosystems and the North American carbon cycle. Glob Change Biol.

[CR66] Paus SM (1997). Die Erdflechtenvegetation nordwestdeutschlands und einiger Randgebiete. Bibl Lichenol.

[CR67] Rola K, Osyczka P (2018). Cryptogamic communities as a useful bioindication tool for estimating the degree of soil pollution with heavy metals. Ecol Indi.

[CR68] Rola K, Osyczka P, Nobis M (2014). Cryptogamic communities dominated by the lichen *Cladonia rei* – a case study of Polish post-smelting dumps in a worldwide context. Herzogia.

[CR69] Strasser RJ, Srivastava A, Tsimilli-Michael M (2000) The fluorescence transient as a tool to characterise and screen photosynthetic samples. In: Yunus M, Pathre U, Mohanty P (eds) Probing Photosynthesis: Mechanisms, Regulation and Adaptation. Taylor & Francis, London, pp. 445e483

[CR70] Sujetovienė G (2010). Road traffic pollution effects on epiphytic lichens. Ekologija.

[CR71] Turpin DH (1991). Effects of inorganic N availability on algal photosynthesis and carbon metabolism. J Phycol.

[CR72] Vannini A, Contardo T, Paoli L, Scattoni M, Favero-Longo SE, Loppi S (2018). Application of commercial biocides to lichens: Does a physiological recovery occur over time?. Int Biodeterior Biodegrad.

[CR73] Van Dobben HF, de Bakker AJ (1996). Re-mapping epiphytic lichen biodiversity in The Netherlands: effects of decreasing SO_2_ and increasing NH_3_. Acta Bot Neerl.

[CR74] Wang M, Wang C, Jia R (2019). The impact of nitrogen deposition on photobiont-mycobiont balance of epiphytic lichens in subtropical forests of central China. Ecol Evol.

[CR75] Wang Y, Yang R, Zheng J, Shen Z, Xu X (2019). Exogenous foliar application of fulvic acid alleviate cadmium toxicity in lettuce (*Lactuca sativa* L.). Ecotoxicol Environ Saf.

[CR76] Yemets O, Gauslaa Y, Solhaug KA (2015). Monitoring with lichens – Conductivity methods assess salt and heavy metal damage more efficiently than chlorophyll fluorescence. Ecol Indic.

