# Supplementary material for: Combined effect of acute salt and nitrogen stress on the physiology of lichen symbiotic partners
Source: Environ Sci Pollut Res Int. 2022 Nov 18;30(10):28192–205. doi: 10.1007/s11356-022-24115-0 (PMC9995433; doi:10.1007/s11356-022-24115-0)
Supplement: Supplementary file 1 — Supplementary file1 (PDF 120 KB) [file 11356_2022_24115_MOESM1_ESM.pdf]

SUPPLEMENTARY MATERIALS TO THE ARTICLE

Combined effect of acute salt and nitrogen stress on the physiology of lichen symbiotic partners

Karolina Chowaniec[1], Anna Żukowska-Trebunia[1], Kaja Rola[1]*

[1] Institute of Botany, Faculty of Biology, Jagiellonian University, Gronostajowa 3, 30-387 Kraków, Poland

* Corresponding author. E-mail address: kaja.skubala@uj.edu.pl (K. Rola)

Table S1 The selected chlorophyll fluorescence (OJIP) derived parameters calculated on the basis of fast fluorescence kinetics used in further analyses

Fluorescence parameter	Description
Basic	
F_0	Minimal fluorescence intensity
F_V/F_M	Maximum quantum yield of PSII photochemistry
The specific energy fluxes per reaction centre (RC)	
ABS/RC	Specific absorption flux per reaction centre
DI_0/RC	Dissipated energy flux per reaction centre
TR_0/RC	Trapped energy flux per reaction centre
ET_0/RC	Electron transport flux per reaction centre
Quantum yields and efficiencies	

Phi (P_0)	Probability that an absorbed photon will be trapped by the reaction centre of PSII
Psi (E_0)	Probability that a trapped exciton moves an electron into the electron transport chain beyond QA
Phi (E_0)	Quantum yield of electron transport
Phi (R_0)	Quantum yield of reduction of end electron acceptors at the PS I acceptor side
Phenomenological energy fluxes per excited cross-section (CS)	
ABS/CS	Specific absorption flux per excited cross-section
DI_0/CS	Dissipated energy flux per excited cross-section
TR_0/CS	Trapped energy flux per excited cross-section
ET_0/CS	Electron transport flux per excited cross-section
Performance index	
PI_{ABS}	Performance index (potential) for energy conservation from photons absorbed by PSII to the reduction of intersystem electron acceptors

Table S2 The results of two-way analysis of variance for the effect of 'salt' and 'nitrogen' factors on *EC* parameter for *Cladonia rei* from unpolluted site (Bukowno town) and polluted site (Trzebinia town)

Study site	Independent variables	F	p	df	Error df	R^2
Unpolluted site	Salt	56.80	<0.001	4	100	0.82
	Nitrogen	28.80	<0.001	4		
	Salt × Nitrogen	6.61	<0.001	16		
Polluted site	Salt	109.26	<0.001	4	100	0.87
	Nitrogen	11.43	<0.001	4		
	Salt × Nitrogen	10.12	<0.001	16		

Table S3 The results of two-way analysis of variance for the effect of 'salt' and 'nitrogen' factors on the F_V/F_M parameter for *Cladonia rei* collected from unpolluted site (Bukowno town) and polluted site (Trzebinia town)

Time after treatment	Study site	Independent variables	F	p	df	Error df	R^2
1h	Unpolluted site	Salt	128.64	<0.001	4	125	0.83
		Nitrogen	14.23	<0.001	4		
		Salt × Nitrogen	2.36	0.004	16		
	Polluted site	Salt	52.99	<0.001	4	125	0.67
		Nitrogen	4.10	0.004	4		
		Salt × Nitrogen	1.73	0.049	16		
24h	Unpolluted site	Salt	35.69	<0.001	4	125	0.64
		Nitrogen	2.02	0.095	4		
		Salt × Nitrogen	4.33	<0.001	16		
	Polluted site	Salt	41.79	<0.001	4	125	0.73
		Nitrogen	28.19	<0.001	4		
		Salt × Nitrogen	3.71	<0.001	16		
72h	Unpolluted site	Salt	24.25	<0.001	4	125	0.68
		Nitrogen	22.43	<0.001	4		
		Salt × Nitrogen	4.77	<0.001	16		
	Polluted site	Salt	6.56	<0.001	4	125	0.69
		Nitrogen	53.72	<0.001	4		
		Salt × Nitrogen	2.65	0.001	16		
